# A Case of Significant Coagulopathy Due to Vitamin K Deficiency Caused by the Administration of Cefazolin and Rifampicin and Hyponutrition After a Postoperative Infection of the Lumbar Spine

**DOI:** 10.7759/cureus.64076

**Published:** 2024-07-08

**Authors:** Masaki Tatsumura, Mikiro Kato, Kei Takahashi, Toru Funayama

**Affiliations:** 1 Department of Orthopaedic Surgery and Sports Medicine, Tsukuba University Hospital Mito Clinical Education and Training Center, Mito Kyodo General Hospital, Mito, JPN; 2 Department of Infectious Diseases, University of Tsukuba, Tsukuba, JPN; 3 Department of Orthopaedic Surgery, University of Tsukuba, Tsukuba, JPN

**Keywords:** cefazolin, abnormal coagulation, coagulopathy, methyl-thiadiazole thiol group, rifampicin, protein induced by vitamin k absence or antagonist-ii, hyponutrition, post-operative infection, vitamin k deficiency

## Abstract

Postoperative surgical site infection in the lumbar spine is one of the serious complications that sometimes results in death. Herein, we describe a case in which a patient was found to have coagulopathy due to vitamin K deficiency when he was transferred to a hospital for treatment for a postoperative infection of the lumbar spine. The coagulation disorder was caused by antimicrobial agents administered to the patient, who was suffering from hyponutrition.

The patient was a 70-year-old man with a history of diabetes mellitus. He was diagnosed with lumbar spinal canal stenosis and underwent posterior decompression of the L2-L5 and S1 laminae at a previous hospital five months before transfer to our hospital. Four months before transfer, purulent discharge was observed from the wound, and methicillin-susceptible *Staphylococcus aureus* was detected in the wound culture. Cefazolin was administered for two weeks, resulting in initial improvement. However, one month before the transfer, the wound infection recurred, accompanied by bacteremia and a psoas abscess. He had been treated with cefazolin, levofloxacin, rifampicin, trimethoprim, and sulfamethoxazole, but the antibiotics' effects were insufficient. Upon transfer for debridement surgery due to uncontrolled infection, his coagulation parameters were as follows: prothrombin time (PT) 74.0 sec, PT-international normalized ratio (INR) 6.69, PT% 9.0, activated partial thromboplastin time (APTT) 138 sec, fibrinogen (FIB) 664 mg/dl, fibrin degradation products (FDP) 7.1 μg/ml, and protein induced by vitamin K absence-II (PIVKA-II) 34400 mAU/ml. Because we suspected vitamin K deficiency, vitamin K 40 mg was administered as a test dose, and coagulation function improved to PT 16.4 sec, PT-INR 1.30, PT% 65.2, and APTT 79 sec after four hours. The diagnosis of vitamin K deficiency was confirmed, vitamin K was administered for four days, and the coagulation status normalized five days after transfer. Debridement was performed for the left psoas abscess. Cefazolin was administered for eight weeks, and administration was completed. The coagulation abnormality did not recur due to careful attention to his nutrition.

We experienced a case of coagulopathy due to vitamin K deficiency caused by antimicrobial agents administered to a hyponourished patient with a postoperative infection of the lumbar spine. The cause of vitamin K deficiency, in this case, was thought to be low nutrition, suppression of vitamin K-producing bacteria by cefazolin and rifampicin, and the use of cefazolin with a methyl-thiadiazole thiol group. It should be kept in mind that severe coagulopathy due to vitamin K deficiency caused by antimicrobial treatment with hyponutrition can occur in postoperative infections.

## Introduction

The incidence of lumbar spinal canal stenosis in Japan is about 9.3% [1], and it is a common disease in the elderly. Posterior decompression is one of the standard procedures for lumbar spinal canal stenosis, and the incidence of postoperative infection for lumbar spinal canal stenosis is 2.1%~3.1% [2]. The most common organism causing postoperative wound infection is *Staphylococcus aureus*, followed by *Staphylococcus epidermidis* [3]. Delayed treatment of postoperative lumbar infections can progress to serious infections such as iliopsoas abscesses and epidural abscesses. Because a serious postoperative infection is often complicated by osteomyelitis of the vertebrae, the duration of antimicrobial therapy for postoperative infection is similar to that for pyogenic spondylitis. For pyogenic spondylitis, a minimum of six weeks of antimicrobial therapy is recommended [4]. If the infection is difficult to control with only antimicrobial agents, such as abscess formation, which leads to the general condition deteriorating, surgical treatment may be necessary to save the patient's life [5].

In addition, the hyponutrition of the patient deteriorates due to difficulty in food intake as a result of the deterioration of the general condition. We report a case of postoperative infection of the lumbar spine with significant abnormal coagulation caused by vitamin K deficiency due to antibiotic agents and hyponutrition.

## Case presentation

**Figure 1:**
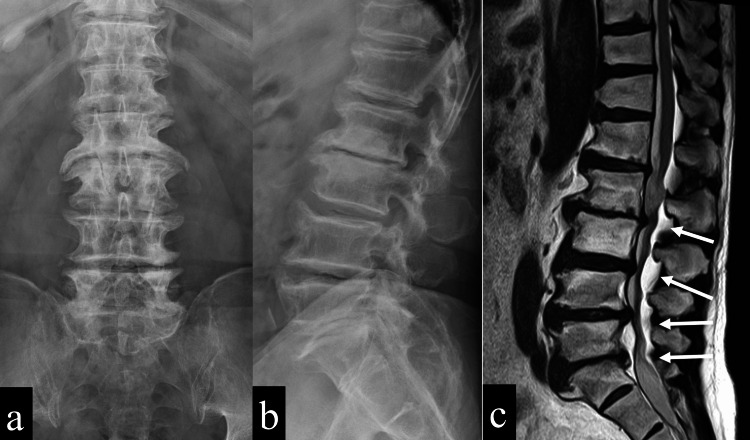
The patient's preoperative images a: A plain x-ray (anteroposterior view) of the lumbar shows multiple bony spurs and no osteolysis.; b: A plain x-ray (lateral view) of the lumbar shows multiple bony spurs and no osteolysis.; c: An MRI (T2-weighted, sagittal view) of the lumbar shows multiple canal stenosis (white arrows).

The patient was a 70-year-old man with a history of diabetes mellitus and benign prostatic hyperplasia. He was working as a contractor just before the surgery for lumbar canal stenosis. He was diagnosed with lumbar spinal canal stenosis (Figure 1) and underwent posterior decompression with partial laminectomy between the second lumbar and first sacrum at the previous hospital five months before transfer to our hospital. 

**Figure 2:**
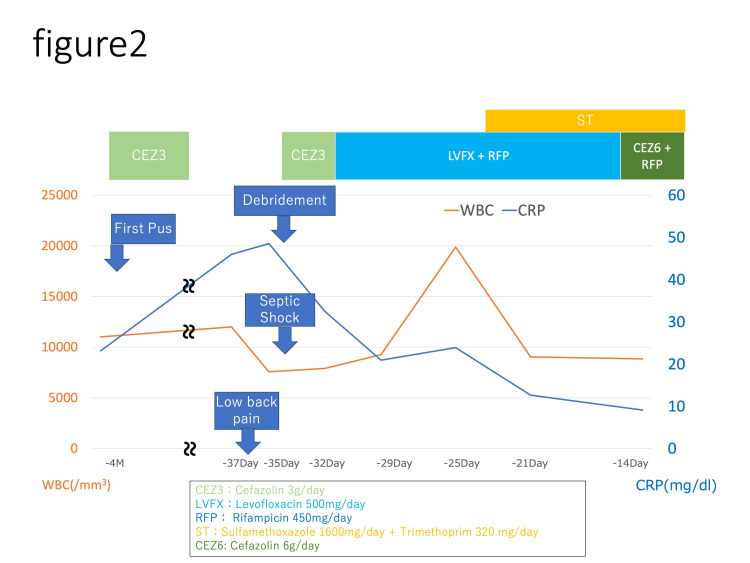
Changes in white blood cell (WBC) and C-reactive protein (CRP) before the patient's transfer to our hospital

Purulent discharge was found to drain from the wound four months before transfer to our hospital, and methicillin-susceptible *Staphylococcus aureus* was detected in the wound culture. The patient was treated with cefazolin 3 g/day for two weeks in the previous hospital, and no additional antimicrobial therapy was given. However, his back pain worsened. He visited the previous hospital and was readmitted 37 days before transfer to our hospital. Blood biochemistry tests revealed a white blood cell (WBC) count of 12000/mm^3^, C-reactive protein (CRP) of 45.97 mg/dl, and serum albumin (Alb) of 2.9 g/dl. Blood culture revealed methicillin-susceptible *Staphylococcus aureus* and the patient was diagnosed as having a postoperative infection with bacteremia. Cefazolin 3 g/day was started, but the patient developed septic shock the day after readmission. Emergency debridement of the lumbar surgical site was performed 35 days before transfer to our hospital, but complete debridement was not achieved due to the extensive extent of the abscess. After readmission, the patient was treated with cefazolin (3 g/day) for seven days, and he developed mucous and bloody stools. His antibiotics were changed to levofloxacin 500 mg/day, rifampicin 450 mg/day, trimethoprim 320 mg/day, and sulfamethoxazole 1600 mg/day for 14 days due to the side effects of cefazolin. After the mucous and hematochezia disappeared, he was treated with cefazolin 6 g/day, rifampicin 450 mg/day, trimethoprim 320 mg/day, and sulfamethoxazole 1600 mg/day (Figure 2, Table 1).

**Table 1:** Changes in white blood cell (WBC), C-reactive protein (CRP), and serum albumin (Alb) before the patient's transfer to our hospital.

Day	-37	-35	-32	-29	-25	-21	-14
CRP	45.97	48.52	32.49	20.94	23.9	12.63	9.08
WBC	12010	7570	7900	9250	19860	9030	8830
Alb	2.9	2.2		2.3		2.5	2.5

From the time of readmission to the previous hospital until transfer to our hospital, the patient had a loss of appetite, and intake of food continued to decline, so intravenous fluids were administered. Because of an uncontrollable extended epidural abscess and an iliopsoas abscess, the patient was transferred to our hospital for additional treatment.

**Figure 3:**
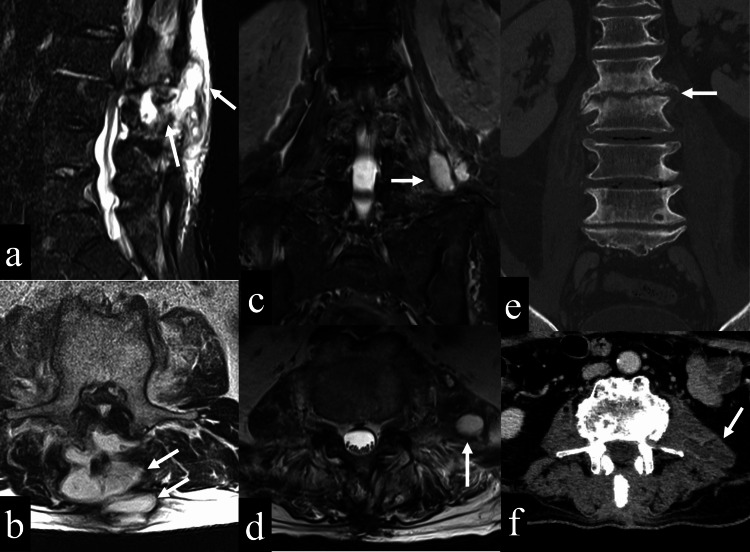
Images after postoperative infection a: An MRI short tau inversion recovery (STIR) (sagittal view) shows the abscess spreading from epidural to subcutaneous tissue (white arrows); b: An MRI (T2-weighted (T2WI), axial view, L2 level) shows the abscess spreading from epidural to subcutaneous tissue (white arrows); c: An MRI STIR (coronal view) shows left psoas abscess (white arrow); d: An MRI (T2WI, axial view, L4 level) shows left psoas abscess (white arrow); e: A CT (coronal view) shows endplate destruction between L2 and L3 (white arrow); f: An enhanced CT (axial view, L4 level) shows left psoas abscess (white arrow).

At the time of the initial examination in our hospital, the patient was 163 cm tall and weighed 60 kg. He could not keep sitting and standing due to low back pain. There was residual numbness in both legs, although there was no motor paralysis. His weight before lumbar surgery was about 75 kg, and he looked gaunt during the initial presentation at our hospital. The MRI showed posterior decompression between the second lumbar and the first sacrum, with bone marrow edema in the vertebral bodies of the second and third lumbar vertebrae and an epidural abscess. Contrast-enhanced CT showed left psoas abscess and endplate destruction between the second/third lumbar (Figure 3). We diagnosed osteomyelitis of the second and third lumbar, an epidural abscess, and a left psoas abscess.

The first treatment plan was to perform debridement surgery after transfer. But the first blood test in our hospital revealed a WBC count of 5000/mm^3^, a CRP of 12.95 mg/dl, and an Alb of 2.7 g/dl. As for the coagulation system, his blood test revealed the following: prothrombin time (PT) 74.0 sec, PT-international normalized ratio (INR) 6.69, PT% 9.0, activated partial thromboplastin time (APTT) 138 sec, fibrinogen (FIB) 664 mg/dl, fibrin degradation products (FDP) 7.1 μg/ml, and D-dimer 1.5 μg/ml. Since significant coagulation abnormalities were observed, it was determined that a thorough examination was necessary, and the scheduled debridement surgery was canceled. The acute disseminated intravascular coagulation (DIC) score was only one point, which was negative for DIC. Liver and renal function were within the standard range. To search for the cause, protein induced by vitamin K absence or antagonist-II (PIVKA-II) was measured and found to be high at 34400 mAU/ml.

**Figure 4:**
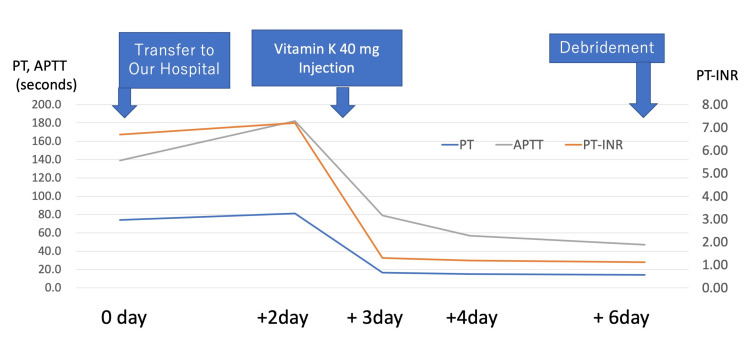
Coagulation test changes around vitamin K dosage We administered vitamin K after coagulation testing on day two. PT: prothrombin time; APTT: activated partial thromboplastin time; INR: international normalized ratio

Suspecting vitamin K deficiency, we administered 40 mg of vitamin K as a test dose, and coagulation function improved to PT: 16.4 sec, PT-INR: 1.30, PT%: 65.2, and APTT: 79.0 sec after four hours (Figure 4). Since the diagnosis of vitamin K deficiency was confirmed, vitamin K was administered for four days (Table 2). We also considered the patient's nutritional status, and he took the supplemental foods suggested by the nutritionist. 

**Table 2:** Coagulation function changes in response to vitamin K administration We administered vitamin K after coagulation testing on day two. PT: prothrombin time; INR: international normalized ratio; APTT: activated partial thromboplastin time

Day	0	2	3	4	6
PT	74.0	81.0	16.4	15.1	14.2
PT-INR	6.69	7.19	1.30	1.19	1.12
APTT	138.8	181.9	79.0	56.7	47.0

**Figure 5:**
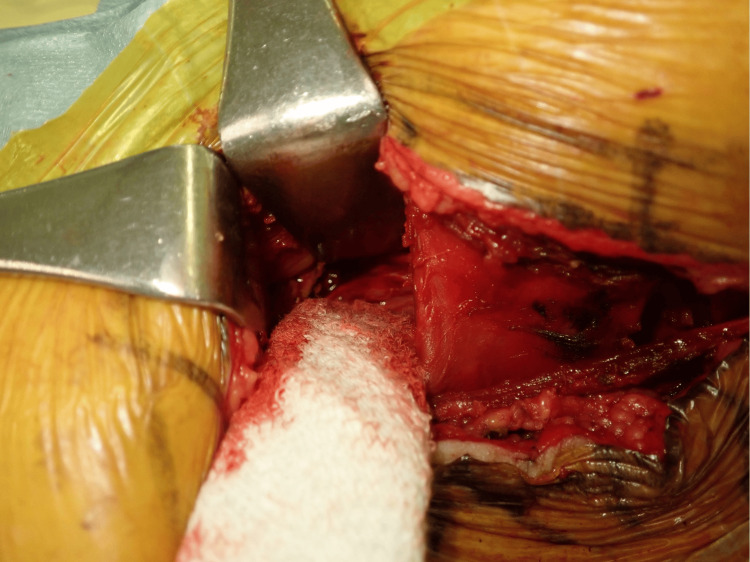
Intraoperative view shows left psoas abscess

Debridement was performed only for the left psoas muscle abscess, which appeared to be refractory six days after transfer to our hospital (Figure 5). Because the epidural abscess was small and did not compress the dural sac with many neurologic symptoms, we decided to treat it conservatively with antimicrobial therapy instead of surgical treatment.

**Figure 6:**
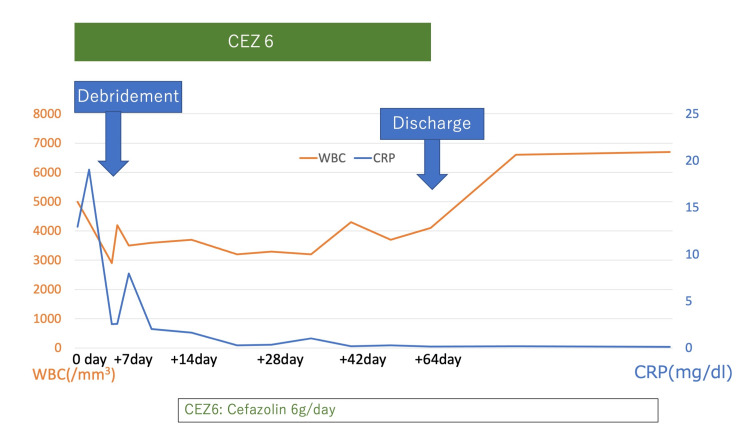
Changes in white blood cell (WBC) and C-reactive protein (CRP) after the patient's transfer to our hospital

The patient was discharged from the hospital after the completion of administration of cefazolin (6 g/day) for eight weeks, as his general condition had improved (Figure 6, Table 3).

**Table 3:** Changes in white blood cell (WBC), C-reactive protein (CRP), serum albumin (Alb), and prealbumin (PAlb) after the patient's transfer to our hospital

Day	0	2	6	7	9	14	21	28	35	42	49	64	71	85	113
CRP	12.95	19.05	2.55	2.59	7.97	2.02	1.64	0.28	0.34	1.03	0.19	0.29	0.14	0.2	0.12
WBC	5000	4300	2900	4200	3500	3600	3700	3200	3300	3200	4300	3700	4100	6600	6700
Alb	2.7	2.4	2.5	2.2	2.3	2.2	2.7	2.9	2.9	3	3.2	3.4	3.4	3.9	4
PAlb		5.2				11.7	12.2	15.3	15.1						

He had recovered to a body weight of 75 kg, was able to walk without a cane, and the numbness had resolved nine weeks after his transfer to our hospital. There was no recurrence of infection or coagulation abnormalities.

**Figure 7:**
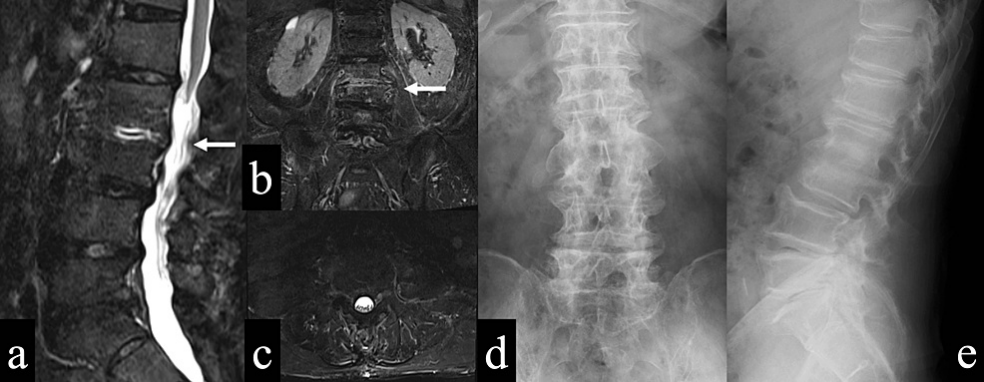
Images three years after the treatment a: An MRI short tau inversion recovery (STIR) (sagittal view) shows slight bone marrow edema of L2/3 (white arrow); b: An MRI STIR (coronal view) shows slight bone marrow edema of L2/3 (white arrows); c: An MRI STIR (axial view) shows no epidural abscess; d: A plain X-ray (anteroposterior view) of the lumbar shows bony fusion of L2/3; e: A plain X-ray (lateral view) of the lumbar shows bony fusion of L2/3.

Laboratory findings were as follows: WBC count 6900/mm^3^, CRP: 0.10 mg/dl, Alb 4.0 g/dl, PT 11.4 sec, PT-INR 0.93, PT% 112.8, and APTT 31.6 sec one year after surgery. The last observation was three years after surgery. Imaging findings included an MRI showing residual bone marrow edema in the second/third lumbar spine, but the iliopsoas abscess had resolved (Figure 7).

## Discussion

In this study, we experienced a case of postoperative infection of the lumbar spine that resulted in a significant coagulation disorder caused by vitamin K deficiency due to the administration of antibiotics in hyponutrition. Although vitamin K deficiency due to cefazolin administration for spontaneous pyogenic spondylitis has been reported [6], vitamin K deficiency due to cefazolin administration for postoperative spinal infection is very rare.

Many antibiotics list vitamin K deficiency as a potential side effect, and it is a common cause of coagulation abnormalities during antibiotic treatment. For the diagnosis of vitamin K deficiency, diagnostic therapy is very common. If PT and APTT improve within two to four hours after intravenous vitamin K administration, the patient is considered to have responded to the trial, confirming the diagnosis of vitamin K deficiency. In this case, PT and APTT were significantly improved following vitamin K administration, thereby confirming the diagnosis. 

The three possible causes of vitamin K deficiency in this patient are (i) a marked decrease in vitamin K intake due to poor nutrition; (ii) decreased vitamin K production due to changes in the intestinal microflora; and (iii) impairment of the vitamin K reduction cycle.

However, vitamin K deficiency is very rarely caused by any of the causes mentioned above because the daily requirement of vitamin K is very low. Multiple causes of these were required to develop significant vitamin K deficiency, resulting in coagulation disorder.

Poor nutrition is one of the most common causes of vitamin K deficiency [7]. In the present case, the patient had poor nutritional intake due to inadequate intake of hospital food as a result of sustained postoperative infection and septic shock. Additionally, the COVID-19 outbreak made the previous hospital prohibit anyone, even the family, from visiting the patient during hospitalization, and no one could bring his favorite food to him. The patient had lost 20% of his body weight during the first visit to our hospital compared to his weight before the surgery for canal stenosis. His weight loss indicated that his food intake was inappropriate, suggesting that his vitamin K intake had decreased.

Decreased vitamin K production due to changes in the intestinal microflora is also a cause of vitamin K deficiency [8]. Normal intestinal microflora produce adequate vitamin K. However, antibiotic treatment decreased the intestinal microflora, changed the intestinal microflora, or shifted to non-vitamin K-producing bacteria. Some bacteria, such as *Staphylococcus aureus*, *Escherichia coli*, and *Bacteroides *spp*.*, have been reported as vitamin K-producing bacteria [8]. Cefazolin has been reported to have antibacterial activity against *Staphylococcus aureus* and *Escherichia coli*, although some *Escherichia coli* are resistant to cefazolin. Rifampicin has been reported to have antibacterial activity against *Bacteroides *spp.. In this case, the patient had been treated with a combination of cefazolin and rifampicin at the previous hospital. This may have decreased the number of vitamin K-producing bacteria such as *Staphylococcus aureus*, *Escherichia coli*, and *Bacteroides *spp*.*, leading to vitamin K deficiency.

Disruption of the vitamin K reduction cycle is another cause of vitamin K deficiency [9]. A factor that can lead to vitamin K deficiency is that antibiotics themselves can impair the vitamin K reduction cycle.

Antibiotics, including N-methyl tetrazole thiol (NMTT) groups, are well-known as cephalosporin antibiotics that interfere with the vitamin K reduction cycle [9]. Conversely, antibiotics including a methyl-thiadiazole thiol (MTD) group, such as cefazolin, are known to disrupt the coagulation cycle [9]. The MTD group has also been shown to inhibit coagulation factor activation [9]. As the use of first-generation cephalosporins has increased with the spread of antimicrobial guidelines, an increasing number of cases have been reported in which the MTD group inhibits the activation of coagulation factors [6, 10]. In the present case, cefazolin, including MTD groups, was also used for treatment.

The causes of vitamin K deficiency, in this case, were considered to be (i) prolonged low nutritional status for more than one month, (ii) suppression of vitamin K-producing bacteria by treatment with cefazolin and rifampicin in the past week, and (iii) the use of cefazolin, including MTD groups. We speculate that the patient's coagulation was not evaluated at the previous hospital, which focused on infection control. This patient developed severe coagulopathy. It is important to recognize that severe coagulopathy due to vitamin K deficiency caused by antimicrobial administration can occur not only in postoperative infections but also in spontaneous infections. As a preventive measure, coagulation tests should be performed periodically during antimicrobial therapy associated with anorexia to monitor for purpura due to bleeding tendency.

## Conclusions

In this study, we reported a case of postoperative infection of the lumbar spine that resulted in vitamin K deficiency due to the administration of antibiotics in a patient with hyponutrition. In this case, vitamin K deficiency was attributed to low nutrition, suppression of vitamin K-producing bacteria with cefazolin and rifampicin, and the use of cefazolin, including MTD groups. It should be kept in mind that severe coagulopathy due to vitamin K deficiency caused by antibiotic therapy can occur in postoperative infections.
